# Beyond emotional regulation: Expanding the existential dimensions of emotional labor in end-of-life care

**DOI:** 10.1017/S1478951526103174

**Published:** 2026-07-09

**Authors:** Faqihul Muqoddam, Yudho Bawono, Oktavian Ismail Johansyah

**Affiliations:** 1 Department of Psychology, Universitas Trunodjoyo Madura, Bangkalan, Indonesia; 2 Department of Psychology, Universitas Airlangga, Surabaya, Indonesia

Dear Editor,

We read with great interest the article by Li et al. (2026). The study makes an important contribution to the literature on emotional labor among healthcare professionals working in end-of-life care. Using a mixed methods approach, the authors provide valuable insights into how palliative care practitioners manage emotional demands, maintain emotional authenticity, establish professional boundaries, and navigate family and cultural dynamics in clinical practice.

One of the major strengths of this article is its successful positioning of emotional labor within the cultural context of Hong Kong, particularly in relation to Chinese cultural values such as death-related taboos, family-centered decision-making, and expectations surrounding emotional expression. The findings further demonstrate that emotional labor in end-of-life care extends beyond surface acting and involves more complex forms of deep acting and authentic emotional engagement.

Nevertheless, we believe that the existential dimension of emotional labor could be further developed as a central conceptual perspective. Although the article comprehensively discusses emotional regulation and professional adaptation, the existential implications of repeated exposure to death, suffering, and loss remain relatively implicit. In end-of-life care settings, healthcare professionals are not only required to regulate professional emotions during clinical interactions but are also continuously confronted with issues related to mortality, helplessness, spiritual suffering, and human vulnerability. Such experiences may shape how professionals understand compassion, spirituality, professional identity, and reflections on their own mortality.

This perspective is consistent with Chan et al. (2016), who emphasized that understanding how palliative care professionals cope with death-related challenges may provide important implications for professional training. Emotional labor, therefore, may be understood not merely as an occupational demand but also as a psychological and existential experience.

The article also effectively highlights the influence of cultural context on emotional labor. However, the dimensions of spirituality and existential meaning-making may deserve greater attention. Bäckersten et al. (2024) argued that healthcare professionals can contribute to existential care through appropriate responsiveness, even within secular societies. Similarly, Tornøe et al. (2014) demonstrated that existential care may emerge through emotional connectedness, sensitivity to patients’ suffering, silence, conversations, and spiritual support.

From this perspective, emotional labor in palliative care may be expanded into a form of “existential labor.” Such a framework may help explain why some healthcare professionals experience emotional exhaustion, whereas others develop resilience, compassion satisfaction, existential maturity, or deeper professional meaning. Kelly and McLeod (2025), He et al. (2023), and Chen and Chen (2024) collectively suggest that repeated exposure to death may simultaneously generate emotional distress, professional growth, greater acceptance of death, and stronger meaning-making capacities among healthcare professionals.

Overall, this article provides an important contribution to understanding the complexity of emotional labor in end-of-life care. Integrating existential and spiritual perspectives into future research may further enrich understanding of how healthcare professionals not only regulate emotions but also construct meaning through repeated encounters with death and human suffering.

Sincerely,
